# Modern Prospection for Hepatic Arterial Infusion Chemotherapy in Malignancies with Liver Metastases

**DOI:** 10.1155/2013/141590

**Published:** 2013-04-17

**Authors:** Yi-Hsin Liang, Yu-Yun Shao, Jia-Yi Chen, Po-Chin Liang, Ann-Lii Cheng, Zhong-Zhe Lin

**Affiliations:** ^1^Department of Hemato-Oncology, E-Da Hospital, No. 1, Yi-da Road, Jiaosu Village, Yanchao, Kaohsiung 82445, Taiwan; ^2^Department of Oncology, National Taiwan University Hospital, No. 7, Chung-Shan South Road, Taipei City 10002, Taiwan; ^3^Graduate Institute of Oncology, College of Medicine, National Taiwan University, No. 1, Section 1, Ren-Ai Road, Taipei City 10051, Taiwan; ^4^Department of Medical Imaging, National Taiwan University Hospital, No. 7, Chung-Shan South Road, Taipei City 10002, Taiwan; ^5^Department of Internal Medicine, National Taiwan University Hospital, No. 7, Chung-Shan South Road, Taipei City 10002, Taiwan

## Abstract

Malignancy with liver metastasis plays an important role in daily oncology practice, especially for primary cancers of the gastrointestinal tract and hepatopancreatobiliary system. On account of the dual vascular supply system and the fact that most metastatic liver tumors are supplied by the hepatic artery, hepatic artery infusion chemotherapy (HAIC) is an appealing method for the treatment of liver metastases. Herein, we summarize recent study results reported in the literature regarding the use of HAIC for metastatic liver tumors, with special focus on colorectal cancer.

## 1. Introduction

Malignancy with liver metastasis plays an important role in daily oncology practice, especially for primary cancers of the gastrointestinal (GI) tract and hepatopancreatobiliary system [1]. The liver is commonly the first site of distant metastasis. For example, about three-quarters of patients with stage IV colorectal cancer (CRC) have liver metastases [2]. Many of these patients have metastatic disease confined to the liver only. It has been demonstrated that for patients with such limited distant metastases, locoregional therapy such as surgery may be helpful [3, 4]. However, usually the liver metastases are too advanced to be resected by hepatectomy. Fewer than 15% of these patients receive hepatectomy to a curative extent [5].

On account of the dual vascular supply system and the fact that most metastatic liver tumors are supplied by the hepatic artery [6, 7], hepatic artery infusion chemotherapy (HAIC) is an appealing method for the treatment of liver metastases. HAIC has several advantages over intravenous chemotherapy. First, chemotherapeutic agents can be delivered more specifically to malignant cells. Normal hepatocytes that mostly rely on the portal venous system are thus exposed to fewer chemotherapeutic agents. Second, many chemotherapy agents used in HAIC have a high first-pass hepatic clearance effect, such as 5-fluorouracil (5-FU) and floxuridine (FUDR), a prodrug of 5-FU. Over 90% of FUDR and 19%–50% of 5-FU are cleared by the liver when they are administered by HAIC [8]. Systemic exposure to chemotherapeutic agents is thus decreased.

These two mechanisms enable HAIC to provide a higher exposure of chemotherapy to malignant cells with minimized toxicities. The higher drug level may also overcome drug resistance. For example, intravenous (IV) anthracyclines are generally considered ineffective for CRC. HAIC with pirarubicin, an anthracycline that is an analogue of doxorubicin, has been demonstrated to have a fair efficacy in CRC patients with liver metastases [9–11]. 

The equipment and skills related to HAIC have been in development for more than 5 decades. With advances in implantable catheters and ports, external infusion pumps can be avoided to decrease catheter-related complications. Catheter implantation is generally performed via the femoral, axillary, or subclavian arteries under fluoroscopic guidance [12–15]. The angiography should be carefully reviewed before and after catheter implantation to identify any anomalous vasculature. The tips of HAIC catheters are fixed at the gastroduodenal artery or proper hepatic artery. The HAIC ports are then immobilized subcutaneously. Finally, a perfusion scan is usually performed for HAIC catheters to detect any unexpected shunting to other organs.

Adverse reactions to HAIC can be divided into catheter-related complications and chemotherapy-related complications. Common catheter-related complications include catheter displacement, hepatic artery occlusion, and catheter-related infection [16–18]. The complication rates for these issues have been reported to be lower than 7% in recent studies, compared to 22–35% in earlier studies. The most common chemotherapy-related complication is gastrointestinal symptoms. Nausea and vomiting can occur in 25–35% patients [17, 19]. Hepatobiliary toxicity, including elevation of serum hepatic transaminase levels, and hyperbilirubinemia are also important problems [20–23]. 

Although the rationale for the use of HAIC for metastatic liver tumors is appealing, the actual benefit of HAIC is not wholly clear. The lack of large randomized clinical trials makes it difficult to examine the overall survival benefits. However, results from previous studies are accumulating gradually and could provide some hints as to the actual efficacy of HAIC for metastatic liver tumors. Herein, we summarize recent study results reported in the literature with regards to the use of HAIC for metastatic liver tumors, with special focus on CRC. 

## 2. Colorectal Cancer

### 2.1. HAIC Combined with Systemic Chemotherapy

CRC is the third most prevalent malignant disease around the world [24, 25]. Despite screening and early surgery, many patients eventually suffer from metastatic disease. The liver is the most frequent metastatic site of CRC. CRC with liver metastasis becomes an important issue for treatment of metastatic CRC, and HAIC potentially provides good local control with a response rate (RR) ranging from 34% to 92% (Table 1) when combined with systemic chemotherapy.

Mancini et al. conducted a clinical trial that enrolled 123 CRC patients with unresectable liver metastasis [27]. The patients were randomized into two arms. In arm one, patients received intravenous 5-FU chemotherapy and infusional cisplatin via HAIC. In arm two, patients received intravenous 5-FU chemotherapy and bolus cisplatin via HAIC. There was no significant difference in response between the two arms, and thus, treatment response was presented as a combination of all patients in the two arms. The overall RR was 52%, which included a 17% complete response rate. The median overall survival (OS) was 18 months and 28 months for all patients and responders, respectively. Kemeny et al. conducted a clinical trial that enrolled 49 patients with CRC who had unresectable liver-confined metastasis only [31]. The patients received intravenous oxaliplatin and irinotecan (CPT-11) combined with FUDR via HAIC. The overall RR was 92%, which included a 8% complete response rate [31]. The median OS was 50.8 months for chemotherapy-naïve patients and 35 months for previously-treated patients. 

Although these studies did not incorporate targeted therapy agents, the reported response rates are comparable to current standards using combination therapy with targeted and cytotoxic chemotherapy. However, whether the addition of HAIC to current standard treatment, which generally provides a high response rate of 47%–64%, is useful remains unclear [32–35]. Recently, targeted therapy has also been used with HAIC in some small series. Bouchahda et al. demonstrated that HAIC could be combined with intravenous cetuximab in two patients in a retrospective study [36]. Further research with different combinations of novel targeted therapy is warranted.

### 2.2. Reversing Inoperable Disease to Operable Disease

For patients with CRC and liver-only metastatic disease, complete resection provides the chance of a cure. When liver metastatic disease develops, complete resection can provide a potential cure for CRC patients. However, only 10–15% of these patients are eligible for such surgery upon diagnosis [5]. Because of the high response rate, HAIC may reverse inoperable liver metastatic disease to an operable status. 

Kemeny et al. conducted a clinical trial examining the use of intravenous oxaliplatin and CPT-11 combined with FUDR via HAIC for patients with CRC-related unresectable liver-confined metastasis only [31]. Initially, 98% of these resectable cases had bilobar metastatic lesions, and 73% of them had >5 hepatic lesions. The overall RR was high at 92%. Twenty-three (47%) patients eventually received hepatectomy to a curative extent. Yamaguchi et al. conducted a clinical trial that enrolled 22 patients who had CRC and unresectable liver metastasis to receive intravenous CPT-11 with oral tegafur/uracil in combination with 5-FU via HAIC [26]. The definition of unresectability included (1) tumors involved all liver segments, (2) inadequate liver reservation after resection, and (3) tumors involved all main hepatic veins or both inflow pedicles. The overall RR was 86.4%, and eventually 14 patients (63.6%) underwent complete resection of liver tumors.

Other than 5-FU, oxaliplatin and CPT-11 have also been tested in HAIC. Ducreux et al. conducted a clinical trial that enrolled 28 patients who had CRC-related inoperable liver-confined metastatic diseases only [37]. Twenty-one of these patients had received previous intravenous 5-FU therapy. The patients then received intravenous 5-FU and leucovorin (LV) with oxaliplatin infused via HAIC. The RR was 64%, and the median overall survival (OS) was 27 months. Approximately 18% of patients' diseases became operable following therapy. The same group then further applied this regimen in a second-line setting [38]. Boige et al. conducted a clinical trial that enrolled 44 patients who had CRC-related inoperable liver-confined metastatic diseases only and in whom first-line chemotherapy failed [38]. Treatment of twenty-eight of these patients with 5-FU, oxaliplatin, and CPT-11 had previously failed. For only one patient, 5-FU alone failed, and in the others both 5-FU and either CPT-11 or oxaliplatin treatment failed. The RR was 62% and the median overall survival (OS) was 16 months. Similarly, 18% of patients' disease became operable following therapy.

For HAIC, the hepatic resectability conversion rate is worthy of emphasis. The current standard treatment, combined targeted and cytotoxic chemotherapy, usually generates a less than 10% hepatic resectability conversion rate according to post hoc analysis [32, 35, 39]. Folprecht et al. conducted the CELIM study that enrolled 114 patients who had CRC and inoperable liver-confined metastatic diseases who received intravenous cetuximab and combination cytotoxic chemotherapy [40]. Overall, 38% of patients eventually received curative hepatectomy. It is worthy of note that 32% of patients with paired images before and after surgery in CELIM trial were considered operable prior to chemotherapy when the images were reviewed centrally. Although there has been no large-scale phase III trial to prove the concept that HAIC might improve the liver resectability conversion rate, the above results are promising, with high response rates and good conversion rates of reversing inoperable disease to operable disease. The outcome is even more encouraging considering that these studies did not incorporate novel targeted agents, such as bevacizumab and cetuximab. 

### 2.3. Liver-Confined Disease

Some patients who receive local therapy for early CRC may suffer from recurrence, with liver metastasis as the only disease site (liver-confined disease). Although systemic chemotherapy is the standard treatment for metastatic CRC, some of these patients had had their primary cancer treated previously and suffered from liver-confined metastatic disease. For these patients, it is reasonable to develop a local therapy with an enhanced efficacy against liver metastases. The mechanism of HAIC suits this purpose. 

HAIC exhibits a high RR, ranging from 22% to 92%, albeit with an unclear effect on overall survival in this setting (Table 2). In the CALGB 9481 trial, 135 CRC patients with inoperable liver-confined disease were randomly assigned to receive FUDR via HAIC or intravenous bolus 5-FU and LV [41]. Patients who received HAIC compared to patients who received IV chemotherapy had a significantly higher RR (47% versus 24%, *P* = 0.012) and a longer median OS (24.4 months versus 20.0 months; *P* = 0.003). Patients treated with HAIC had a significantly longer time to hepatic progression compared to patients who received IV chemotherapy (9.8 months versus 7.3 months; *P* = 0.034), but a significantly shorter time to extrahepatic progression (7.7 months versus 14.8 months; *P* = 0.029).

Besides FUDR or 5-FU, mitomycin-C (Mit-C) has also been used in HAIC [47, 49]. Kemeny et al. randomly assigned 63 CRC patients with inoperable liver-confined disease to receive high-dose Mit-C and FUDR/LV via HAIC [47]. The RR was 73% in the chemotherapy-naïve patients and 70% in previously-treated patients. However, the expense was a high biliary toxicity. Elevation of the serum bilirubin level >3 mg/dL occurred in 22.5% patients. Half of the patients suffered from at least a doubling of the serum hepatic transaminase level. Besides, biliary sclerosis was noted in 6 patients (9.5%) and liver bilomas in 5 (7.9%) patients. 

Fallik et al. enrolled 75 patients with CRC and inoperable liver-confined metastatic diseases in a phase II trial [11]. All patients received intravenous 5-FU and LV in combination with HAIC using pirarubicin, an anthracycline analog. The overall RR was 31.9% and the median OS was 19 months. Most important was that grade 4 neutropenia was reported for 27 cycles (23%). The toxicity profile seemed acceptable in this trial and no cardiac toxicity was reported.

Several small studies in the literature have addressed the use of HAIC in liver-confined disease of CRC. The study designs and results were heterogeneous across these trials. Therefore, Mocellin et al. conducted a meta-analysis to compare HAIC and intravenous chemotherapy for liver-confined metastatic diseases of CRC [54, 55]. Ten randomized controlled trials, including a total of 1277 patients, were enrolled in the analysis. All studies used 5-FU or FUDR as single agents via HAIC or intravenous chemotherapy. Although the RR was significantly higher in patients receiving HAIC than in patients receiving intravenous chemotherapy (42.9% versus 18.4%, *P* < 0.001), the median OS was not significantly longer (15.9 months versus 12.4 months, *P* = 0.240). 

The result of this meta-analysis should be interpreted cautiously. The analyzed ten clinical trials were mostly conducted a decade ago and used 5-FU only in intravenous chemotherapy, which is clearly inadequate as compared with present therapies. This explains the inferior OS outcome of 12 months only in either treatment arm and the uncertainty regarding the interpretation of this meta-analysis result. On the contrary, many patients who were allocated into the HAIC arms in these trials did not receive HAIC mainly due to catheter-related complications. Some of them were allowed to cross over into intravenous chemotherapy arms but still analyzed as HAIC in an intent-to-treat manner. All these reasons suggest difficulty in interpretation of this meta-analysis. 

According to current evidence, HAIC demonstrates better locoregional control for CRC patents with liver-confined disease, at the expense of poor extrahepatic disease progression. Although there was a survival benefit for HAIC-treated patients reported in the CALGB 9481 study, this OS benefit became nonsignificant when ten studies were enrolled into a meta-analysis. Evidence as to whether HAIC provides a better survival benefit than systemic therapy is thus still lacking, and further large-scale clinical trials are warranted. Except for 5-FU and FUDR, some other cytotoxic agents such as Mit-C and pirarubicin are also applied via HAIC. As we know, anthracycline drugs were thought to be ineffective in the treatment of CRC. However, anthracycline analogs demonstrated potential efficacy in CRC via HAIC because of their special mechanism, which provides a greater drug selection for the treatment of CRC.

### 2.4. HAIC after Curative Hepatectomy

With improvement in surgical techniques, more and more CRC patients with liver-confined metastasis receive surgery for both the primary CRC and liver tumors with a curative intent. Prevention of disease recurrence is crucial in these patients. Some physicians use a local treatment, HAIC, in this adjuvant setting. 

Kemeny et al. enrolled 156 CRC patients who received complete resection of liver metastatic disease [56, 57]. These patients were randomized into groups receiving either intravenous 5-FU alone or in combination with HAIC using FUDR. In an updated result after a median follow-up duration of 10.3 years, patients who received combination therapy with HAIC had a significantly longer progression-free survival than patients who received intravenous therapy alone (31.3 months versus 17.2 months, *P* = 0.02) [57]. Although the OS was not significantly different, the trend still favored combination therapy with HAIC (68.4 months versus 58.8 months, *P* = 0.10). 

Oxaliplatin is the current standard for adjuvant treatment of stage III CRC [58]. When the efficacy of newer agents for the treatment of CRC has been proven, they have been tested for HAIC. Alberts et al. conducted a phase II trial that enrolled 76 patients with CRC who had liver-confined metastasis [59]. After curative surgery for both the primary tumor and liver metastases, patients received adjuvant intravenous oxaliplatin and oral capecitabine alternated with HAIC FUDR plus dexamethasone. Although 3 treatment-related deaths were reported, the median disease-free survival was 32.7 months and only 30 patients developed recurrent malignancies after median follow-up time of 4.8 years.

In addition to oxaliplatin, CPT-11 has also shown a fair efficacy for stage 4 CRC and thus was also examined in combination with HAIC. Kemeny et al. conducted a phase I/II trial that enrolled 96 patients with CRC who had liver-confined metastasis [60]. After curative surgery, patients received adjuvant intravenous CPT-11 combined with HAIC FUDR plus dexamethasone. With a median follow-up time of 26 months, the 2-year survival rate was 89%, and 1.5-year hepatic disease-free survival rate was 88%. 

In the targeted therapy era, a combination of HAIC with novel targeted agents was also tested in some series. Kemeny et al. randomly assigned 73 CRC patients with resected liver-confined disease to receive curative hepatectomy [61]. All patients received intravenous oxaliplatin or CPT-11 plus infusional 5-FU in combination with FUDR plus dexamethasone via HAIC. Patients were randomized into two arms, receiving intravenous bevacizumab or not. The 4-year recurrence-free survival rate was 46% and 37% for the no bevacizumab arm and the bevacizumab arm (*P* = 0.4), respectively, after a median follow-up duration of 30 months.

There have recently been some convincing results showing a lower recurrence rate for HAIC in combination with systemic therapy after curative hepatectomy. Systemic chemotherapy after curative surgery for liver metastatic disease is still the standard treatment, and HAIC might provide enhanced locoregional control for the liver. Further large-scale phase III trials are warranted. 

## 3. Other Malignancies

### 3.1. Gastric Cancer

The prognosis of gastric cancer with liver metastases is extremely poor, with median OS of only 2–6 months if untreated [62]. The standard treatment is combination systemic chemotherapy including platinum analogs and 5-FU. For better palliation, some case series reported the efficacy of HAIC as a liver-directed therapy. Tarazov reported the results of HAIC using 5-FU and doxorubicin in 12 patients with unresectable gastric cancer-related bilobar liver metastases [62]. The RR was 25% and the median OS was 23 months. One patient had 60 months of stable disease after 7 courses of HAIC treatment. Kumada et al. conducted a phase II trial that tested HAIC with 5-FU, epirubicin, and Mit-C in 63 patients with gastric cancer who had unresectable liver metastasis [63]. Only 36 patients were documented to have liver-confined metastatic diseases. The response rate was 55.6%, with three complete responders. For patients with liver-confined disease, the median OS was 13 months.

As a treatment for synchronous multiple liver metastases from gastric cancer after palliative gastrectomy to maintain quality of life, Ojima et al. retrospectively analyzed 18 patients who received HAIC with 5-FU [64]. The RR was 83% with a response duration of 7.6 months. The median OS was 19.2 months. 

According to the limited data above, HAIC potentially provides high response rates in patients with liver metastases of gastric cancer. The median OS in these small groups of patients seemed longer given that the best survival in patients receiving systemic chemotherapy has been reported to be 13.8 months [65]. Therefore, HAIC might have the potential to be a feasible local treatment for gastric cancer with unresectable liver metastases.

### 3.2. Uveal Melanoma

Uveal melanoma usually hematogenously spreads into the liver in up to 95% patients [66]. Once liver metastases occur, the life expectancy is less than 5 months. Because no systemic therapy is proved to have definite efficacy for metastatic uveal melanoma, regional therapy to control liver metastases and delay extrahepatic spread becomes one of the treatment choices. Melichar et al. performed HAIC with the combination of cisplatin, vinblastine, and dacarbazine in 10 liver metastatic uveal melanoma patients [67]. Two patients had partial response, and four patients achieved stable disease. Those who had clinical benefit survived for more than one year. Becker et al. conducted a phase II prospective clinical trial that enrolled 48 patients with metastatic uveal melanoma [66]. HAIC with fotemustine was given to the 23 patients who had liver metastases alone. Intravenous fotemustine was given to the 25 patients who had metastases other than liver. All patients received subcutaneous interleukin-2 and interferon *α*. The overall RR was significantly higher for patients who received HAIC than those patients who received intravenous chemotherapy (21.7% versus 8.0%). However, the median OS was similar (369 days versus 349 days). 

With the therapeutic activity demonstrated above, HAIC might play a role to control liver-confined metastatic uveal melanoma. Comparing with the cumulating results from chemoembolization in uveal melanoma with liver metastases, the evidence for the better efficacy of HAIC is still scarce and needs more studies [68].

### 3.3. Pancreatic Cancer

The prognosis of pancreatic cancer is extremely poor because of the low resection rate at diagnosis, rapid progression, and frequent metastasis even after curative surgery. Despite the advances of cancer therapy generally, the survival of patients with pancreatic cancer did not improve significantly in the past decades. Liver is the most common site of metastasis, and thus, HAIC was examined as a strategy for palliation or prevention of liver metastasis. For unresectable pancreatic cancer without metastasis, HAIC was also examined as a primary treatment modality for primary tumors from pancreatic body and tails [69]. 

Homma et al. also enrolled 16 patients with pancreatic cancer-related liver metastases who received cisplatin and 5-FU via HAIC [70]. The RR was 68.8% with median survival 16.25 months.

There were few studies focusing on HAIC in the adjuvant setting. Hashimoto et al. conducted a retrospective analysis that enrolled 42 patients with pancreatic cancer who received curative pancreatectomy and subsequent 5-FU via HAIC [71]. Hepatic recurrence rate was 7.1% with a median 19-month followup. 

From the above studies, HAIC for pancreatic cancer is a way for local treatment. Besides, HAIC also provides potential benefit to reduce recurrence after pancreatectomy, compared to the 36% recurrent rate reported by CONKO-001 study using systemic gemcitabine alone [72]. The cost of relative high complication rate remains the problem. Common complications include high probability of hepatic arterial stenosis (19.6%) and liver abscess (3.6%). Due to the limitation of various HAIC techniques and different vasculatures of each patient, large prospective trial is required for further investigation.

### 3.4. Biliary Tree Cancer

Due to limited effective therapy for unresectable and metastatic biliary tract cancers, HAIC was also applied in several studies. These studies of biliary tract cancer were heterogeneous in patient population, and most studies included more than one cancer type. Inaba et al. conducted a phase I/II trial for patients with unresectable intrahepatic cholangiocarcinoma [73]. HAIC with gemcitabine was applied in 13 patients. One patient had partial response and 8 patients had stable disease. The response rate was 7.7%. In addition to gemcitabine, cisplatin and epirubicin combination were also examined. Cantore et al. conducted a phase II study that enrolled 25 patients with metastatic intrahepatic cholangiocarcinoma and 5 patients with gallbladder carcinoma to receive intravenous 5-FU and HAIC with cisplatin and epirubicin [74]. Overall RR was 40% including 1 patient who achieved complete remission. Median OS was 13.2 months. Mambrini et al. conducted a phase II trial that enrolled 20 patients with unresectable metastatic intra- or extra-hepatic biliary tree cancers to receive oral capecitabine and HAIC using cisplatin and epirubicin [75]. The overall RR was 31.5%, and median OS was 18 months. 

From the evidence of these phase II studies, combination HAIC with oral or intravenous chemotherapy seems to be a safe and effective treatment modality. With the advance of intervention radiology and radiotherapy techniques, multimodality treatment incorporating radiation, drug-eluting beads, and chemoembolization were also developing in combination with HAIC [76, 77]. Further comparison of different treatments modality and large scale phase III trials are needed.

### 3.5. Neuroendocrine Tumor

Gastroenteropancreatic neuroendocrine tumors often metastasize to liver and contribute substantially to one of the most important noncolorectal causes of liver metastases [78]. Due to limited patients numbers, HAIC for unresectable liver metastases from neuroendocrine tumors has mostly been studied retrospectively. 

Christante et al. collected 77 patients with extensive liver metastases with disease progression after octreotide treatment [79]. Fifty-nine patients received four cycles of 5-FU via HAIC with the addition of selective chemoembolization at the end of third and fourth cycles. However, 18 patients received HAIC alone due to the concern of hepatotoxicity. Overall response rate was 80%, and median progression-free survival was 19 months, and all the responders were treated with combination of HAIC and chemoembolization.

Most of the HAIC studies in neuroendocrine tumors were conducted in combination with chemoembolization. Due to the limitation of scarce retrospective studies with HAIC treatment alone, the efficacy of HAIC for neuroendocrine tumors seems to be difficult to clearly be identified based on current evidences. Further studies are warranted. 

## 4. Conclusion

In this article, we presented the current lines of evidence of HAIC as a treatment of liver metastases. HAIC provides a good locoregional control to liver tumors. Most of evidences mainly came from studies of CRC. For patients with CRC and inoperable liver metastasis, HAIC has potentials to enhance the treatment response of the liver metastases when combined with systemic chemotherapy. For CRC patients who had failed previous intravenous chemotherapies, HAIC still provides fair efficacy of control to liver tumors. Patients with initially considered inoperable liver metastases could have a chance to receive surgery if HAIC converts the tumors back to operable status. However, the evidence to support if HAIC could totally replace the intravenous chemotherapies is still not strong enough across previous trials. Therefore, current standard for liver metastatic CRC is still intravenous chemotherapy, and HAIC could be provided as local control focusing on liver. As for CRC patients with initially operable liver metastatic tumors who received curative operation, HAIC in combination with intravenous chemotherapies demonstrated good competence to reduce liver recurrent and to subsequently prolong the overall survival. Some new agents could be used in HAIC in combination to systemic agents, such as pirarubicin, which is initially considered ineffective for CRC. With the emergent novel agents and targeted agents in the 21 century, more studies are needed for different combinations with HAIC. 

HAIC is also applied for other malignant diseases with liver metastases, especially for those malignancies which have poor response to systemic chemotherapy, such as melanoma or pancreatic cancer. Although the results for large-scale prospective phase III trials are warranted, HAIC seems to become an attractive procedure for hepatic metastatic diseases in the future.

## Figures and Tables

**Table 1:** Selective studies of combining HAIC with systemic chemotherapy for colorectal cancer.

Authors	Year	Setting	Treatment	Line	Inclusion population	Patient no. (treat^a^)	Median OS (months)	RR	Note
Yamaguchi et al. [26]	2011	Pro, phase I/II	HAIC → 5-FU IV → CPT-11 + LV Oral tegafur/uracil	First line	Unresectable hepatic mets	Phase 1: 12 (12) Phase 2: 22 (22)	Not reach	86.4%	RCR: 63.6%

Mancini et al. [27]	2003	Pro, Ran	Arm1: HAIC → continuous cisplatin IV → 5-FU Arm2: HAIC → bolus cisplatin IV → 5-FU	First line	Unresectable hepatic mets	58 (58) 65 (65)	18	52%	

Goéré et al. [28]	2010	Ret	HAIC → oxaliplatin IV → 5-FU + LV	First line: 18 Second line: 69	Unresectable hepatic mets	87 (87)	NM	55%	5-year survival: 56%

Gallagher et al. [29]	2007	Ret	HAIC → FUDR + Dexa IV → CPT-11	Failed oxaliplatin	Unresectable hepatic mets	39 (39)	18	44%	

Pilati et al. [30]	2009	Ret	Arm1: HAIC → FUDR + LV Arm2: HAIC → FUDR + LV IV → 5-FU + LV	NM	Unresectable hepatic mets	72 (72) 81 (81)	18 19.1	52.7% 50.6%	

Selected studies that enroll patients with colorectal cancer to receive systemic chemotherapy in combination with HAIC are listed here. Studies designed for patients with colorectal cancer-related liver-confined metastatic disease were listed in Table 2.

*With statistical significance.

^a^Actual patients' number who received treatment.

Abbreviations—OS: overall survival, RR: response rate, Pro: prospective, Ran: randomized, Ret: retrospective, NM: not mentioned, HAIC: hepatic artery infusion chemotherapy, IV: intravenous, FUDR: floxuridine, LV: leucovorin, Dexa: dexamethasone, CPT-11: irinotecan, Mets: metastasis, and RCR: resectability conversion rate.

**Table 2:** Selective studies of HAIC for liver-confined metastatic disease from colorectal cancer.

Authors/Year	Setting	Treatment	Line	Inclusion population	Patient no. (treat)^a^	Median OS (months)	RR	Note
Kemeny et al. 2006 [41]	Pro, Ran	Arm1: HAIC → FUDR + LV + Dexa Arm2: IV → 5-FU + LV	First line	Unresectable liver confined	68 (59) 67 (58)	24.4* 20	47%* 24%	QOL improvement

Fiorentini et al. 2006 [42]	Pro, phase III	Arm1: HAIC → 5-FU + LV Arm2: HAIC → 5-FU + LV IV → 5-FU + LV	First line	Unresectable liver confined	40 (36) 42 (40)	14 20	41.7% 47.5%	

Fallik et al. 2003 [11]	Pro, phase II	HAIC → pirarubicin IV → 5-FU + LV	First line	Unresectable liver confined	75 (69)	20	34.4%	

Kerr et al. 2003 [43]	Pro, Ran	Arm1: HAIC → 5-FU + LV Arm2: IV → 5-FU + LV	First line	Unresectable liver confined	145 (95) 145 (126)	14.7 14.8	22% 19%	

Allen-Mersh et al. 2000 [44]	Pro, Ran	Arm1: HAIC → FUDR IV → 5-FU + LV Arm2: IV → 5-FU	First line	Unresectable liver confined	41 (39) 43 (42)	NM	45% 23%	No QOL difference

Lorenz et al. 2000 [45]	Pro, Ran	Arm1: HAIC → 5-FU + LV Arm2: IV → 5-FU + LV Arm3: HAIC → FUDR	First line	Unresectable liver confined	57 (40) 57 (71)^b^ 54 (37)	18.7 17.6 12.7	45% 19.7% 43.2%	

Kemeny et al. 2009 [31]	Pro, phase I	HAIC → FUDR + Dexa IV → oxaliplatin + CPT-11	First line: 23 Second line: 26	Unresectable liver confined	49 (49)	First line: 50.8 Second line: 35	92%	RCR: 47%

Ducreux et al. 2005 [37]	Pro	HAIC → Oxaliplatin IV → 5-FU + LV	First line: 7 Second line: 21	Unresectable liver confined	28 (26)	27	64%	RCR: 18%

Kemeny et al. 2005 [46]	Pro, phase I	Arm1: HAIC → FUDR + DEXA IV → oxaliplatin + CPT-11 Arm2: HAIC → FUDR + DEXA IV → oxaliplatin + 5-FU + LV	First line: 4 After first line: 32	Unresectable liver confined	36 (36)	36 22	90% 87%	

Kemeny et al. 2009 [31]	Pro, phase I	HAIC → FUDR + Dexa IV → Oxaliplatin + CPT-11	First line: 23 Second line: 26	Unresectable liver confined	49 (49)	First line: 50.8 Second line: 35	92%	RCR: 47%

Kemeny et al. 2005 [47]	Pro, phase II	HAIC → FUDR + Dexa + Mit-C	First line: 26 Second line: 37	Unresectable liver confined	63 (63)	First line: 23 Second line: 20	First line: 73% Second line: 70%	

Lorenz et al. 2001 [48]	Pro, phase II	HAIC → 5-FU + LV	First line: 40 Second line: 10	Unresectable liver confined	50 (50)	22.3	56%	

Boige et al. 2008 [38]	Pro	HAIC → oxaliplatin IV → 5-FU + LV	After first line	Unresectable liver confined	44 (43)	16	62%	RCR: 18%

Fazio et al. 2003 [49]	Ret	HAIC → cisplatin + Mit-C + 5-FU	After first line	Hepatic mets predominent^c^	45 (44)	NM	35%	

Kemeny et al. 2001 [50]	Pro, phase I	HAIC → FUDR + DEXA IV → CPT-11	After first line	Unresectable liver confined	46 (46)	17.2	74%	

Van Riel et al. 2000 [51]	Ret	HAIC → 5-FU	All	Hepatic mets predominent^c^	145 (145)	14.3 m	34%	Hepatic artery thrombosis (48%)

Fujimoto et al. 2009 [52]	Ret	HAIC → 5-FU	NM	Unresectable liver confined	72 (72)	18	38%	

Sameshima et al. 2007 [53]	Ret	HAIC → 5-FU	NM	Unresectable liver confined	42 (42)	29.1	57%	

Selected studies that enroll patients with colorectal cancer-related liver-confined metastatic disease are listed here. Studies designed for patients with colorectal cancer to receive systemic chemotherapy in combination with HAIC are listed in Table 1.

*With statistical significance.

^a^Actual patients' number who received treatment.

^b^Patients who did not receive treatment in arm1 and arm3 received treatment as arm2.

^c^Trial enrolled patients with liver-confined disease or “minimal” extrahepatic disease.

Abbreviations—OS: overall survival, RR: response rate, Pro: prospective, Ran: randomized, Ret: retrospective, NM: not mentioned, HAIC: hepatic artery infusion chemotherapy, IV: intravenous, FUDR: floxuridine, LV: leucovorin, Dexa: dexamethasone, Mit-C: mitomycin C, CPT-11: irinotecan, QOL: quality of life, Mets: metastasis, and RCR: resectability conversion rate.

## Abbreviations

CRC: Colorectal cancer
HAIC: Hepatic artery infusion chemotherapy
OS: Overall survival
5-FU: 5-Fluorouracil
FUDR: Floxuridine
RR: Response rate
CPT-11: Irinotecan
LV: Leucovorin
TACE: Transarterial chemoembolization
MIT-C: Mitomycin C
IV: Intravenous
GI: Gastrointestinal.
